# Labor Market Consequences of Grandparenthood

**DOI:** 10.15195/v11.a22

**Published:** 2024-08-09

**Authors:** Won-tak Joo, Felix Elwert, Martin D. Munk

**Affiliations:** a) University of Florida;; b) University of Wisconsin-Madison;; c) Halmstad University;; d) Uppsala University

**Keywords:** grandparents, extended family, intergenerational, multigenerational, Denmark, marginal structural models

## Abstract

Little is known about the labor market consequences of becoming a grandparent. We estimate grandparenthood effects on labor supply and earnings using detailed multigenerational data from Danish population registers. Results show that the consequences of grandparenthood are unequally distributed and starkly patterned. Becoming a grandparent reduces hours worked and income, especially for grandmothers, more so when the grandchild is born to a daughter, and most when the grandmother’s daughter gives birth as a teenager. Grandfathers also experience a reduction in hours worked (but not income) from their daughter’s teen birth, but the reduction is much smaller than among grandmothers. The effects of a daughter’s teen birth are further amplified for low-income grandmothers. Our results imply that childbearing has multigenerational consequences that are structured by gendered caregiving, the caregiving needs of the parent generation, and the delegating capacity of the grandparent generation.

The labor market consequences of childbearing extend beyond parents to grandparents. Because parents typically are the primary caregivers, past research has mainly focused on the effects of childbearing on parents. Results show that parenthood increases household labor specifically for mothers (Baxter, Hewitt, and Haynes 2008; Craig and Mullan 2010; Neilson and Stanfors 2014) and reduces the economic participation and wages of women, but not of men (Budig and England 2001; Gangl and Ziefle 2009; Kleven, Landais, and Søgaard 2019; Lundberg and Rose 2000; Vagni and Breen 2021).

More recently, rapid population aging has shifted attention to multigenerational caregiving and the role of grandparents in the family support system (e.g., Bengtson 2001; Cherlin and Furstenberg 1986; Leopold and Skopek 2015). Nevertheless, the implications of grandparenthood for grandparents’ labor market outcomes remain underresearched. A small number of studies show that becoming a grandmother accelerates women’s retirement in the United States (Lumsdaine and Vermeer 2015) and Europe (Backhaus and Barslund 2021; Frimmel et al. 2022; Kridahl 2017), and reduces work hours in the United States (Rupert and Zanella 2018). Estimates for grandfathers are less consistent (Backhaus and Barslund 2021; Kridahl 2017; Rupert and Zanella 2018) and often unexamined (Frimmel et al. 2022; Lumsdaine and Vermeer 2015). Crucially, prior research on grandparenthood exclusively focused on grandparents themselves and hardly considered the role of parents in the production of grandparenthood effects.

Research on grandparenthood is as fruitful sociologically as it is challenging methodologically because it requires theorizing and modeling how multigenerational interactions are framed by the social norms and material constraints of both generations. Because grandparenthood is not initiated by those who experience it (i.e., grandparents) but by their children (Hagestad and Lang 1986), the labor market consequences of grandparenthood are best understood as multigenerational interactions between grandparents and parents.

This study analyzes the labor market consequences of becoming a grandparent using a multigenerational perspective. First, we investigate heterogeneity in grandparenthood effects by the gender of grandparents and parents. Despite evidence for the dominance of the mother-daughter bond in intergenerational relations (Chodorow 1978; Fingerman 2001; Rossi and Rossi 1990; Silverstein and Bengtson 1997), prior research has not investigated how grandparenthood effects vary jointly with the gender of both generations.^1^

Second, we estimate whether grandparenthood effects vary by the age of parent’s childbearing as a proxy for parents’ childcare needs. Specifically, although it is well known that teenage childbearing leads to lasting economic disadvantages for parents (Fletcher and Wolfe 2009; Hynes and Clarkberg 2005; Kane et al. 2013; Miller 2011), its spillover effects on the grandparent generation are unknown.^2^

Third, we examine how grandparents’ economic resources-as proxies for their capacity to delegate caregiving-moderate grandparenthood effects. Previous studies provide inconsistent evidence for the moderating effect of grandparents’ work hours or earnings (Frimmel et al. 2022; Rupert and Zanella 2018). Hence, we examine effect modification by grandparents’ income, jointly with the gender constellation of both the grandparent and parent generations and the timing of grandparenthood.

Our study draws on data of unique scope and resolution. Specifically, we analyze detailed multigenerational records of 15 complete birth cohorts of second generation (G2) “parents” from Danish population registers, track their fertility histories for the birth of third generation (G3) “grandchildren” (if any), and link these to the employment and income histories of first generation (G1) “grandparents” until they reach the statutory retirement age.

We find that the consequences of grandparenthood for working-age grandparents are unequally distributed and starkly patterned. Becoming a grandparent decreases the employment and annual income, particularly of grandmothers and especially when grandchildren are born to grandmothers’ daughters. Although effect sizes for grandmothers are generally modest, effects are substantively large when grandmotherhood is initiated by a daughter’s teenage birth and when grandmothers have limited economic resources. Our results thus suggest that childbearing has multigenerational consequences that are structured by gendered caregiving, the caregiving needs of the parent generation, and the capacity of the grandparent generation to delegate caregiving.

The article proceeds as follows. First, we consolidate past research and formulate hypotheses. Next, we discuss the construction of our analytic data set from multigenerational Danish population registers and explain our causal modeling strategy. Third, we present results for the effects of grandparenthood on labor market outcomes and their dependence on multigenerational family characteristics. We conclude with implications for future research.

## Theoretical Expectations

Becoming a grandparent may affect a grandparent’s labor market outcomes — employment, hours, and income — via multiple mechanisms. Grandparenthood effects likely arise both from grandparents’ desire and the necessity to spend time with grandchildren and to support parents. Desire and necessity, in turn, will be structured by prevailing norms, and the age and socioeconomic circumstances of the parent and grandparent generations, among other factors.

Following prior research, we expect negative effects of grandparenthood on grandparents’ labor market outcomes on average, as grandparents reduce their working hours to spend time with grandchildren (direct grandparenting) or to support their own children in their new role as parents (indirect grandparenting) (Backhaus and Barslund 2021; Frimmel et al. 2022; Kridahl 2017; Lumsdaine and Vermeer 2015). Going beyond prior research, we further expect that grandparenthood effects will depend on the multigenerational circumstances of grandparents and parents. Next, we articulate detailed hypotheses regarding factors that may modify the size of grandparenthood effects focusing on grandparents’ and parents’ gender, parents’ age at having a child, and grandparents’ economic resources.

### Grandparents’ and Parents’ Gender

We expect that gender will modify grandparenthood effects in three respects. First, G2 gender will modify grandparenthood effects because the division of labor inside and outside of the home remains gendered, especially for parents. After child-birth, gender disparities in domestic labor and paid work increase as mothers assume the majority of the parenting tasks (Baxter et al. 2008; Craig and Mullan 2010; Neilson and Stanfors 2014; Offer and Schneider 2011; Raley, Bianchi, and Wang 2012; Sanchez and Thomson 1997) and their labor force participation and wages decrease (Budig and England 2001; England et al. 2016; Kleven et al. 2019). Because G2 mothers shoulder more caregiving tasks and hence require more support, more parenting tasks will spill over to G1 grandparents from their G2 daughters than from their G2 sons (Aassve, Arpino, and Goisis 2012; Arpino, Pronzato, and Tavares 2014; Dimova and Wolff 2011; Geurts et al. 2015; Meyer 2014).

Second, G1 grandparents’ gender likely modifies grandparenthood effects because the gendered allocation of childcare and support also applies to grandparents. G1 grandmothers meet most of the demand for intergenerational care labor as kin keepers who coordinate the circulation of resources through kinship networks (Hagestad 1986; Rosenthal 1985) and spend more time in care work for grandchildren than grandfathers (Di Gessa et al. 2016a; Fuller-Thomson and Minkler 2001; Hank and Buber 2009; Igel and Szydlik 2011; Luo et al. 2012). Active grandparenting by maternal G1 grandmothers at the birth of a G3 grandchild is near universal in contemporary Western societies (Di Gessa et al. 2016a; Geurts et al. 2015; Hank and Buber 2009; Thomese and Liefbroer 2013).^3^

Third, G1 and G2 gender may jointly modify grandparenthood effects to produce especially large effects for G1 grandmothers of G2 daughters, for all the reasons that kindle strong mother-daughter bonds (Rossi and Rossi 1990; Silverstein and Bengtson 1997). For example, as Fischer (1981) argued in view of the empathic foundation of female intergenerational solidarity and grandparenting (Chodorow 1978; Fingerman 2001), childbearing may enhance a G2 daughter’s understanding of her G1 mother’s life, which, in turn, may encourage G1’s grandmothering in the female line.

Hence, we expect that the birth of a G3 grandchild places greater demands on G1 grandmothers than on G1 grandfathers, resulting in decreased economic participation mostly among G1 grandmothers, especially when the G3 grandchild is born to a G2 daughter, and little change for grandfathers.

H1: G2’s childbearing decreases G1’s labor force participation and annual income, especially among G1 grandmothers of G2 daughters.

### Teenage G2 Parenthood

G2 parents’ age at having a G3 grandchild likely affects the demand for G1 grandparental involvement. Older G2 parents have accumulated more human capital and life experiences, enabling them to hold a better-paying job (Hynes and Clarkberg 2005; Miller 2011), to establish a stabler relationship with partners (McLanahan 2004; McLanahan and Jacobsen 2015; Musick 2002), and to have better access to childcare information and services (Fergusson and Woodward 1999; Pogarsky, Thornberry, and Lizotte 2006). Hence, older G2 parents may have less need for G1 grandparental support.

Conversely, G2’s teenage childbearing should increase the demand for G1’s contribution for at least three reasons. First, many G2 teen parents are not fully prepared to parent on their own, as many are enrolled in education, are unmarried or unpartnered, and have limited economic resources (Furstenberg 1980). Second, most teen births are unplanned and hence more disruptive for both G2 parents and G1 grandparents (Furstenberg 1976; Musick 2002). Third, the consequences of teenage childbearing, including loss of education and income, are long-lasting for parents (Fletcher and Wolfe 2009; Hynes and Clarkberg 2005; Kane et al. 2013; Miller 2011), and likely create more demand for grandparental support in the long run.

H2: G2’s childbearing causes a larger decrease in G1’s labor force participation and annual income when G2 gives birth as a teenager.

### Grandparent’s Economic Resources

G1 grandparents may support G2 parents by investing time in assisting G2 parents or providing direct childcare for their G3 grandchild, or by providing funds to purchase substitutes for these services on the market. We expect that G1 grandparents with lower economic resources will experience greater negative effects of grandparenthood on their labor market outcomes for two reasons. First, economic constraints will impel low-income G1 grandparents to provide time rather than money to G2 and G3, hence reducing the time available for staying engaged in the labor market. Conversely, because of the greater opportunity cost of reducing work hours, high-income G1 grandparents may prefer providing monetary support and remain engaged in the labor market. Second, low-income workers often have less flexible work schedules, and thus any increase in G2 parents’ demand for G1 grandparents’ time will be more disruptive for G1’s employment. These hypotheses are aligned with previous findings that coresidential grandparenting is more common among low-income grandparents (Burton 1990; Luo et al. 2012; Meyer 2014; Sarkisian and Gerstel 2004).

H3: G2’s childbearing causes a larger decrease in labor force participation and annual income of G1 with lower income.

## Institutional Context of Grandparenthood in Denmark

We estimate the consequences of grandparenthood in Denmark over 20 years from 1992 to 2012. During this period, the total fertility rate ranged from 1.7 to 1.9; the average age at childbearing increased from 28.7 to 30.9 for women and 31.5 to 33.6 for men; and the teenage birth rate decreased from 9.5 to 3.0 births per 1000 teenage women. The average expected years of life remaining at age 40 increased from 34.6 to 39.0 years for men and from 39.1 to 42.6 years for women, and the statutory retirement age varied between 65 and 67 (Lund and Villadsen 2005).^4^

Denmark provides an interesting test case for grandparent effects because it follows a “defamiliarized” policy regime with extensive public resources for childcare and lower normative pressure for private assistance on family members (Saraceno and Keck 2010). Denmark has supported mothers’ labor force participation through universal childcare since 1964 and paternity leave for both parents since 1984 (Abrahamson 2010), resulting in the best childcare coverage in Europe (Saraceno and Keck 2010). Partly owing to this policy advantage, Denmark enjoys greater gender equality in the division of household labor than, for example, the United States and other European countries (Craig and Mullan 2010; Hook 2010); lower probabilities of multigenerational coresidence; and lower obligations for and less time spent on intergenerational support, including grandchild care (Albertini, Kohli, and Vogel 2007; Di Gessa et al. 2016a; Hank and Buber 2009; Kalmijn and Saraceno 2008). Hence, grandparenthood effects in Denmark likely represent lower bounds on grandparenthood effects elsewhere.

Nonetheless, there are multiple reasons to expect an impact of grandparenthood on grandparents’ labor market outcomes—even in Denmark—and that the effect of grandparenthood will vary by gender and age at childbearing. First, public policy does not completely substitute for transfers within the family: the exchange of support between generations may be less intensive but also more frequent as policy support for child and elder care increases in Europe (Albertini et al. 2007; Hank and Buber 2009). Second, despite public support for female workers, recent research shows that parenthood causes an immediate 30 percent decrease in annual income and a 20 percent decrease in working hours specifically for Danish women, resulting in 21 percent and 7 percent of cumulative declines in income and employment, respectively, over 20 years after childbearing (Kleven et al. 2019). This suggests that traditional women-centered parenting—and hence plausibly also women-centered grandparenting—patterns still hold in Denmark. Third, although teenage childbearing is relatively rare in Denmark, teenage mothers are greatly disadvantaged in their educational and occupational careers (Johansen, Nielsen, and Verner 2020; Robson 2003) compared to women who first become mothers in their early twenties (Rosenbaum 2020). Teenage childbearing is also heavily concentrated among economically and socially disadvantaged families (Christoffersen and Lausten 2009; Knudsen and Valle 2006), suggesting that grandparenthood due to a child’s teenage parenthood affects a particularly vulnerable population.

## Data and Methods

We estimate the labor market consequences of grandparenthood with population-level longitudinal data from Danish population registers. Danish population registers are uniquely suited for this purpose because they allow for tracking multiple generations of families from the 1960s and further provide complete labor market, marriage, education, and health histories from roughly 1980 onward. We link information from multiple registers using unique individual pseudo-identification numbers generated by Statistics Denmark.

### Population

We target all Danish-origin G1 men and women who had at least one biological G2 born in Denmark between 1977 and 1992.^5^ Follow-up begins in the year in which the first G2 born during this period turned 15 years of age. We exclude G1s who did not live continuously in Denmark or whose first G2 had a G3 grandchild before age 15. To eliminate extreme outliers, we also exclude G1 grandparents who ever had an annual income below zero or above one million Danish Kroner (DKK, approximately 170 thousand U.S. dollars), excluding 3 percent of G1.^6^ To focus on the labor market consequences of grandparenthood on grandparents of working age, we censor G1 panels at age 63, before reaching the statutory retirement age. The final analytic sample includes N=889,899 unique G1 grandparents for a total of 12,180,659G1 person-years.

### Grandparenthood

We record the fertility histories of each G1’s first four G2 children born between 1977 and 1992 for 20 years from 1992 until 2012.^7^ The primary treatment variable of our analysis is the start of G1’s grandparenthood, which we define as the birth of each G1’s first G2’s first G3.^8^ Hereafter, “G2” denotes the first G2 born to G1 between 1977 and 1992, and “G3” denotes the first G2’s first G3, unless otherwise noted.

### Labor Market Outcomes

G1’s labor market outcomes are traced from G2’s fifteenth birthday until the end of follow-up in 2012. We focus on three outcomes. First, *degree of employment* is the standard measure of labor market participation, derived from hours worked, in Denmark, ranging from 0 to 1,000. The maximum value represents year-round full-time employment, including legal vacations. Second, an indicator for *full-time employment* equals 1 if the degree of employment exceeds 750, which is achieved when a G1 works more than 27 hours per week year-round. Third, *annual labor income* equals the sum of taxable and tax-free wages, bonus pay, severance pay, and stock options, measured in 2012 DKK.

### Covariates

We control for an extensive set of potential confounding factors for G1’s grandparenthood and labor market outcomes. All covariates are measured individually for each G1 and their first G2, and via summary indices for all of G1’s G2s. We differentiate three groups of covariates. First, *time-fixed covariates* F include G1’s and first G2’s gender, and an indicator for G1’s own teenage childbearing (i.e., giving birth before the age of 20). Second, *time variables* S include G1’s and first G2’s ages and calendar year as sets of one-year dummies. Our primary time variable is first G2’s age, T∈S, which is set to t=1 when G2 is 13 years old. Our analyses trace G1’s labor market outcomes from t=3 (when G2 is 15 years old), and we track control variables from t=1, two years before the earliest occurrence of grandparenthood.

Third, we include four groups of *time-varying covariates* $\bar{V}=\left\{V_{1},\ldots V_{t}\right\}$. (i) G1’s time-varying characteristics include their work experience, marital status, education, and health status. All analytic models include lagged values of all of G1’s labor market variables except for the focal outcome of the model as part of $\overset{‾}{V}$. Work experience is measured by total years of employment. Education is measured by years of education and an indicator of current enrollment. Regarding health status, we control for chronic diseases using the Charlson comorbidity index (Charlson et al. 1987), days of hospitalization, and an indicator for mental health measured by any contact with a mental hospital or prescription of psychoactive medicine. We control for family status (partnered, divorced, widowed, never married) at t=1, and for being partnered (vs. not) afterward, because most transitions after baseline are between partnered and other statuses. We capture G1’s spouse’s or partner’s time-varying characteristics using five annual indicators: full-time employment, labor income ≥300,000 DKK,^9^ any chronic disease, hospitalized ≥5 days, and any mental health contact. Additionally, we control for the total number of G1’s biological children. (ii) We include the same time-varying covariates for G1’s first G2. Importantly, we create a teen birth indicator if the first G2 first gave birth as a teenager (aged 15 to 19). We also add the first G2’s degree employed and labor income as covariates. (iii) For G1’s first four G2s, we control for the total number of G2 and G3, respectively, and the number of G2s who are female, are married, are full-time employed, earn labor income ≥300,000 DKK, have 16 or more years of education, are currently enrolled in school, have any chronic disease, were hospitalized for five or more days per year, or had any mental health contact. (iv) G1’s time-varying household characteristics include the total number of household members, the number of G2s living in the household, the number of household members under age 18, household gross income, and an indicator for receiving any public assistance.

Online supplement Table A1 gives a full list of variables used in this analysis.

### Identification

Our goals are to estimate (i) the causal effects of grandparenthood on G1’s labor market outcomes; (ii) effect moderation in these grandparenthood effects by G1’s gender, G2’s gender, G2’s teen birth, and G1’s economic resources; and (iii) how these effects evolve over the duration of grandparenthood.

Let $Y_{it}$ be the observed labor market outcome of G1 i at time t. Let $D_{it}$ be grandparenthood status, so that $D_{it}=1$ if i is a grandparent at t, and ${\overset{‾}{D}}_{it}=\sum_{s=0}^{t}D_{is}$ be the number of years i has been a grandparent. We define $Y_{it}\left({\overset{‾}{D}}_{it}\right)$ as the time-varying potential labor-market outcome if i had been a grandparent for ${\overset{‾}{D}}_{it}$ years at time t. Then the causal effect of grandparent trajectory ${\overset{‾}{D}}_{it}$ compared to ${\overset{‾}{D}}_{it}^{\prime }$ on labor-market outcome $Y_{it}$ is given by

(1) $$Y_{it}\left({\overset{‾}{D}}_{it}\right)-Y_{it}\left({\overset{‾}{D}}_{it}^{\prime }\right)$$

We capture effect heterogeneity in grandparenthood effects by estimating conditional causal effects. For example, the causal effect of grandparenthood duration ${\overset{‾}{D}}_{it}$ on labor-market outcome $Y_{it}$ may vary with G2’s teen birth $B_{it}$,

(2) $$Y_{it}\left({\overset{‾}{D}}_{it}|B_{it}\right)-Y_{it}\left({\overset{‾}{D}}_{it}^{\prime }|B_{it}^{\prime }\right)$$

where $B_{it}=1$ if i is a grandparent at t due to G2’s childbearing before G2’s age of 20. We note that all effect modifiers B are realized at or before the onset of grandparenthood, so that our conditional potential outcomes are well defined.

Identification of these causal grandparenthood effects is possible under three assumptions (Robins 1999). First, the *consistency* assumption requires that person i’s observed outcome given their actual grandparenthood history equals their potential outcome if they had been assigned the same grandparenthood history by external intervention. Second, we assume *sequential unconfoundedness*: the potential labor market outcome is statistically independent of the observed grandparenthood status given the observed past (i.e., past labor market outcome ${\overset{‾}{Y}}_{ik-1}$, past grandparenthood trajectory ${\overset{‾}{D}}_{ik-1}$, and all other covariates ${\bar{V}}_{ik-1},S$, and F):

(3) $$Y_{it}\left(D_{ik}\right)⊥D_{ik}|{\overset{‾}{Y}}_{ik-1},{\overset{‾}{D}}_{ik-1},{\bar{V}}_{ik-1},S,F,\;t\geq k.$$

In a slight abuse of notation, because grandparenthood is irreversible so that no confounding can take place after G1 has entered grandparenthood, our concern is only with confounding factors up until the initiation of grandparenthood.

**Figure 1:**
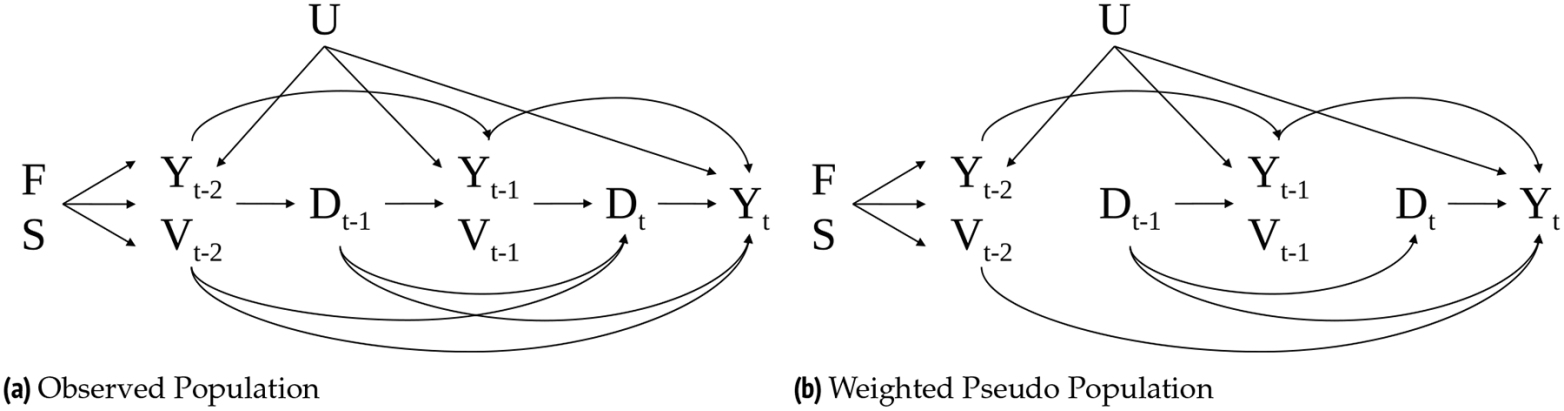
The assumed causal data-generating mechanism for the last two periods of observation (a) and associations remaining after inverse probability weighting (b). Y: labor market outcome; D: grandparenthood; F: time-fixed covariates; S: time trends; V: time-varying covariates; U: unobserved covariates. Arrows represent causal effects.

Figure 1(a) illustrates the assumed data-generating process as a directed acyclic graph, involving grandparenthood D, labor market outcome Y, time-varying covariates V, time trends S, time-fixed covariates F, and unobserved factors U from time t-2 to t. Under the assumption of sequential unconfoundedness, there are no unobserved factors that cause both D and Y. Note, however, that sequential unconfoundedness does allow U (e.g., G1’s cognitive skills) to affect past and future time-varying factors V and Y (Elwert 2013).

Third, we assume *positivity*, also known as *common support*: the probability of being a grandparent is strictly inside the interval [0, 1] for any combination of past covariates, including outcome histories.

### Estimation: Repeated-Measure Marginal Structural Models

Under these assumptions, the key challenge for estimating causal grandparent effects is *dynamic selection* into grandparenthood, in the sense that whether a G1 is not yet a grandparent in one period affects the time-varying confounders that determine whether G1 becomes a grandparent in a subsequent period. This is illustrated in Figure 1(a). Here, whether a G1 is a grandparent at time $t-1\left(D_{t-1}\right)$ influences G1’s employment or welfare receipt at time $t-1\left(Y_{t-1},V_{t-1}\right)$, which, as a measure of G1’s capacity to provide support to G2, influences whether G2 has a child that turns G1 into a grandparent at $t\left(D_{t}\right)$.

We illustrate the implications of dynamic selection for the estimation of grandparenthood effects using directed acyclic graphs (Elwert 2013). The causal effect of ${\overset{‾}{D}}_{t}$ on $Y_{t}$ in Figure 1(a) comprises four causal paths from $D_{t}$ and $D_{t-1}$ to Y (i.e., $D_{t}\to Y_{t},D_{t-1}\to Y_{t},D_{t-1}\to Y_{t-1}\to Y_{t}$, and $D_{t-1}\to V_{t-1}\to Y_{t}$). In conventional single-equation regression models predicting the causal effect of $\overset{‾}{D}$ on $Y_{t}$, researchers usually adjust for the entire observed past, that is, past V, lagged Y,S, and F, to avoid *confounding bias*. However, direct control for those factors leads to two types of bias. First, control for all past Y and V would induce *over-control bias* by blocking causal paths from the past D to the current Y (e.g., $D_{t-1}\to Y_{t-1}\to Y_{t}$). For example, if G2’s childbearing at t-1 causes a decrease in G1’s working hours at t-1, then direct control for degree employed at t-1 would result in the underestimation of the effect of $D_{t-1}$ on degree employed at t. Second, if any unobserved factor U influencing both past and future Y and V exist (e.g., unobserved labor market skills), then past Y and V would be common outcomes of U and D (i.e., collider variables) and direct control for past Y and V would open noncausal paths between U and D (e.g., $D_{t-1}\to V_{t-1}\leftarrow U\to Y_{t}$) and lead to *endogenous selection bias* (Elwert and Winship 2014). Hence, both controlling and not controlling for these time-varying covariates would induce bias.

We address the estimation challenge posed by dynamic selection by adopting a *repeated-measure marginal structural model* (R-MSM) (Hernán, Brumback, and Robins 2002; VanderWeele et al. 2011). R-MSMs indirectly control for time-varying covariates using inverse-probability weights for being a grandparent, which results in the weighted pseudo population, illustrated in Figure 1(b), where past Y and V no longer confound the causal effect of D on Y and hence do not need to be controlled in the outcome models that are estimated on the weighted data. In contrast to conventional marginal structural models (MSMs) used in prior sociological research with a single outcome at the end of follow-up, R-MSMs are appropriate for time-varying outcomes measured across the duration of follow-up.

Estimation of R-MSM proceeds in three steps. First, we predict the probability of becoming a grandparent in each period from very flexibly specified logistic regressions of grandparenthood on the observed past,

(4) $$\begin{array}{c} P\left[D_{t}|{\overset{‾}{D}}_{t-1},{\bar{V}}_{t-1},{\overset{‾}{Y}}_{t-1},S,F\right]=\left\{\begin{array}{rr} \text{logistic}\left(V_{t-1},V_{t-2},Y_{t-1},Y_{t-2},S,F\right), & \text{if}\;D_{t-1}=0\\ 1, & \text{if}\;D_{t-1}=1 \end{array}\right. \end{array}$$

where the probability of grandparenthood for those who have already become a grandparent in a prior period is set to 1. Probabilities are predicted from a pooled logistic regression model that regresses grandparenthood $D_{t}$, using all G1 person-years where G1 had not yet become a grandparent from t=3 to 23 (i.e., G2 from age 15 to 35) on once- and twice-lagged time-varying covariates $V_{t-1},V_{t-2},Y_{t-1},Y_{t-2}$; time variables S; and time-fixed covariates, F. We include V at t=1 in F, except for G1’s and G2’s current educational enrollment, first G2’s labor market outcomes (because hardly any are in the labor force at baseline), and composite indices for the first four G2 (because their baseline summary characteristics are almost perfectly determined by first G2’s characteristics). We include the time variables in S as yearly dummy variables for T (i.e., G2’s age), G1’s age, and calendar year to allow for nonlinear trends. We enter all variables as main effects, and we additionally interact all variables with the G1-G2 gender constellation (i.e., three dummies for male–female, female–male, female–female, using male-male as the reference category) and all covariates except time variables S with linear and quadratic terms of T to allow for gender- and age-specific trends of grandparenthood.^10^ By permitting an extremely flexible specification of time trends and interactions with gender and age, our models account for the social process of G2’s childbearing that is strongly gendered and evolves as G2 gets older.

We compute time-varying inverse probability of treatment weights, $WD_{t}$, from the probabilities predicted in Equation (4),

(5) $$WD_{t}=\prod_{k=3}^{t}\frac{P\left[D_{k}|D_{k-1},D_{k-2},S,F\right]}{P\left[D_{k}|D_{k-1},D_{k-2},V_{k-1},V_{k-2},Y_{k-1},Y_{k-2},S,F\right]}$$

where the numerator is obtained from logistic regressions that are analogous to those in Equation (4) but excluding time-varying covariates and lagged outcomes. Under maintained assumptions, these weights remove the dependence of the potential outcomes on the time-varying covariates and past outcome histories.^11^

Second, to correct for possible bias due to selective attrition, we calculate attrition weights, $WA_{t}$. Attrition is limited in our data, as only 9.1 percent of G1s were excluded from the sample before the end of follow-up in 2012. We code attrition $A_{t}=1$ when (i) G1 emigrates (1.2 percent); (ii) G1 (4.6 percent), G2 (0.5 percent), or G3 (0.2 percent) dies; or (iii) any missing value exists in $Y_{t},D_{t},V_{t-1}$, or $Y_{t-1}$ (2.7 percent; mostly related to temporary breaks in the G2 panel [V], likely due to temporary emigration).^12^ We do not model exits due to the definition of the study population (i.e., the calendar year is >2012; G1’s age is >63) because they are exogenous. We treat death, emigration, and missingness as the same attrition process. We estimate the probability of $A_{t}$ based on $D_{t-2},V_{t-2},Y_{t-2},S$, and F using logistic regression, allowing interactions between all variables and G1–G2 gender and between $D_{t-2},V_{t-2},Y_{t-2}$, and linear and quadratic terms of T.^13^ Attrition weights at each period t are given by

(6) $$WA_{t}=\prod_{k=3}^{t}\frac{P\left[A_{k}|D_{k-2},S,F\right]}{P\left[A_{k}|D_{k-2},V_{k-2},Y_{k-2},S,F\right]}.$$

The final time-varying stabilized weight is given by the product of the exposure and attrition weights:

(7) $$W_{t}=WD_{t}\times WA_{t}.$$

Third, we weight the data by $W_{t}$, effectively creating a pseudo population in which past Y and V no longer confound the outcome Y and D. Thus, we can estimate the causal effect of grandparenthood without direct control for the past Y and V in the outcomes model.

We estimate several models separately by G1’s gender, and jointly by G1’s and G2’s gender, to capture gender-specific effects of grandparenthood:

(8) $$Y_{t}=\beta_{0}+\beta_{1}D_{t}+\beta_{2}D_{t}B_{t}+f(S,F)+\varepsilon .$$

First, our main R-MSM of Equation (8) estimates the effect of present grandparenthood, $D_{t}$, on G1’s labor market outcome, $Y_{t}$, and how this effect depends on G2’s teenage parenthood, $B_{t}$. Because $B_{t}$ is always zero when $D_{t}$ is zero, the equation omits the main-effect term for $B_{t}$. Our interest is in the effect of G2’s non-teen birth, $\beta_{1}$, and in the effect of G2’s teen birth, $\beta_{1}+\beta_{2}$ on G1’s labor market outcomes. The function f(S,F) contains baseline covariates S and F used in the estimation of numerators and denominators of the stabilized final weights, $W_{t}$, including main-effects terms for all elements of S and F, and interaction terms between F and linear and quadratic terms of T.

Second, to evaluate the trajectory of grandparenthood effects, we additionally estimate a version of Equation (8) that allows the effects of grandparenthood to vary freely by the duration of grandparenthood across the grandchild’s first 10 years of life.

Third, we test effect modification by two baseline moderators—G1’s labor income and household income when G2 was aged 13 to 15, each divided into tertiles—by performing separate analyses for each subsample.

We estimate all models using weighted least squares (WLS) regression with stabilized weights and cluster-robust standard errors at the level of the individual (Hernán et al. 2002; VanderWeele et al. 2011). Because we analyze a very large sample, we adopt a stringent α=0.01 criterion for statistical significance. All tests are two-sided.

For comparison, we also estimate four more traditional models: (a) unweighted ordinary least squares (OLS) regression controlling only for time trends; (b) unweighted OLS regression with controls for time trends and time-invariant covariates; (c) unweighted OLS regression controlling for time trends, time-invariant covariates, and time-varying covariates at t-1; and (d) individual-level fixed-effects regression with time trends, and time-varying covariates at t-1. Results for these models are shown in Appendix A of the online supplement.

## Results

### Descriptive Statistics

Online supplement Table A2 reports descriptive statistics measured at G2’s age 13 (t=1) and 25 (t=13) separately by G1 and G2’s gender. We observe the usual descriptive differences in G1’s labor market outcomes by G1’s gender—but not by G2’s gender—when G2 is 13 years of age. The gender gap in degree employed narrows with age and becomes negligible by the time G2 is aged 25 and has presumably left the parental home, whereas the gender gap in labor income remains unchanged. G1’s grandparenthood by G2’s age 25 is more common when G2 is a daughter (21 percent) than when G2 is a son (11 percent), reflecting that women on average have children at an earlier age than men. Overall, 327,104 (36.8 percent) out of the 889,899 G1s in our sample became grandparents during the study period, and 19,341 (2.2 percent) became grandparents because of their G2 son’s or daughter’s teenage birth.

Online supplement Table A3 shows descriptive statistics for the stabilized weights. The weights are centered around zero across all G1–G2 gender subsamples. Following standard practice, we trim large weights above 14.0 to reduce the influence of a small number of extreme cases (Hernán et al. 2002; Sharkey and Elwert 2011), which results in the change of only 658 G1-years’ weights.

### Grandparenthood Effects by Gender and G2’s Teen Birth

**Figure 2:**
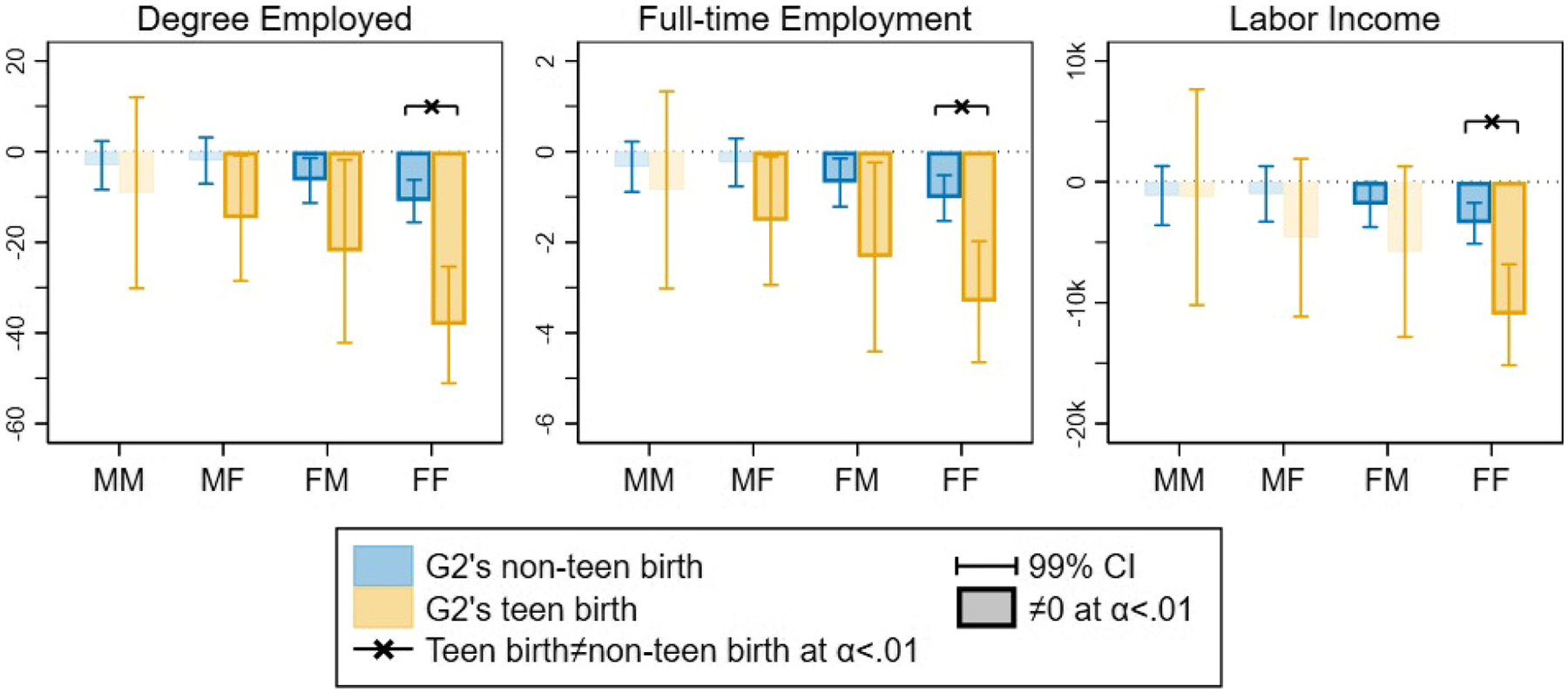
Grandparenthood effects on grandparent’s labor market outcomes by G1 and G2 gender and G2’s teen birth. Estimates from Table 1. MM: male G1–male G2; MF: male G1–female G2; FM: female G1–male G2; FF: female G1–female G2.

The estimated causal grandparent effects on G1’s three labor market outcomes from G2’s non-teen and teen birth by G1 and G2’s gender combination are visualized in Figure 2 (the corresponding R-MSM coefficients from Eq. [8] are presented in Table 1).

Figure 2 shows that grandparenthood effects are patterned by G1’s and G2’s gender and by G2’s teen birth across all three labor market outcomes. Becoming a grandparent decreases the degree to which G1 is employed, especially for G1 grandmothers and most when the G3 grandchild is born to a G2 daughter. The estimated effect of G2 daughter’s non-teen birth on G1 grandmothers is highly statistically significant (p value < 0.001) but substantively modest in size. Compared with their average degree employed before the start of grandparenthood, G1 grandmothers experience a $-\frac{10.9}{689.4}=-1.6$ percent decrease in their degree of employment after G2 daughter’s non-teen birth. By contrast, the effect of becoming a G1 grandmother due to a G2 daughter’s teen birth on the grandmother’s degree of employment is substantively large at $-\frac{\left(10.9+27.3\right)}{548.3}=-7$ percent. Grandparenthood reduces the degree of employment for grandfathers only if their G2 daughter (but not their son) gives birth as a teenager, but the size of the reduction is less than half of the case for grandmothers with G2 daughter’s teen birth.

Results for grandparent effects on full-time employment show the same patterns. Grandparenthood reduces the probability of full-time employment and the amount of annual labor income for G1 grandmothers, more so when G3 is born to a G2 daughter rather than to a G2 son, and especially when the G2 daughter gives birth as a teenager. Again, the estimated causal effect of grandmotherhood on full-time employment is highly statistically significant, substantively small when the G2 daughter gives birth as an adult, at $-\frac{1.0}{64.8}=-1.6$ percent, and substantively large when the G2 daughter gives birth as a teenager, at $-\frac{\left(1.0+2.3\right)}{49.5}=-6.7$ percent. Grandfathers experience a reduction in full-time employment only when a G2 daughter (not a son) gives birth to G3 as a teenager (not as an adult), and the effect is smaller than for grandmothers.

Finally, grandparenthood due to G2 daughter’s non-teen birth reduces G1 grandmothers’ annual labor income slightly by $-\frac{3430}{240524}=-1.4$ percent, although grandmotherhood due to a G2 daughter’s teen birth reduces G1 grandmothers’ income substantially by $-\frac{\left(3430+7565\right)}{167602}=-6.6$ percent compared to their average income before becoming a grandmother. There is no detectable effect of grandparenthood on a grandfather’s income.

In sum, these results confirm hypotheses H1 and H2. Regarding H1, we find that G2’s childbearing has the largest effect on all three labor market outcomes for G1 grandmothers of G2 mothers. Regarding H2, we find that becoming a grandparent because of G2’s teenage childbearing does indeed lead to a larger decrease in labor force participation and annual income for all grandparents, but the difference in effects is only statistically significant for G1 grandmothers of G2 mothers (Figure 2).

Online supplement Tables A4–S6 show supplementary estimates from conventional regression models (without inverse probability weighting) and fixed-effects models. As expected, regression models that do not adjust for time-varying covariates produce larger grandparent-effect estimates than our preferred R-MSM estimates, likely because they neglect time-varying confounding. Conversely, conventional regression models that do control for time-varying covariates, just like individual-level fixed-effects models, yield smaller estimates than our preferred R-MSM estimates, likely because they overcontrol for time-varying covariates that lie on the causal pathway from past grandparenthood status to outcomes.

**Figure 3:**
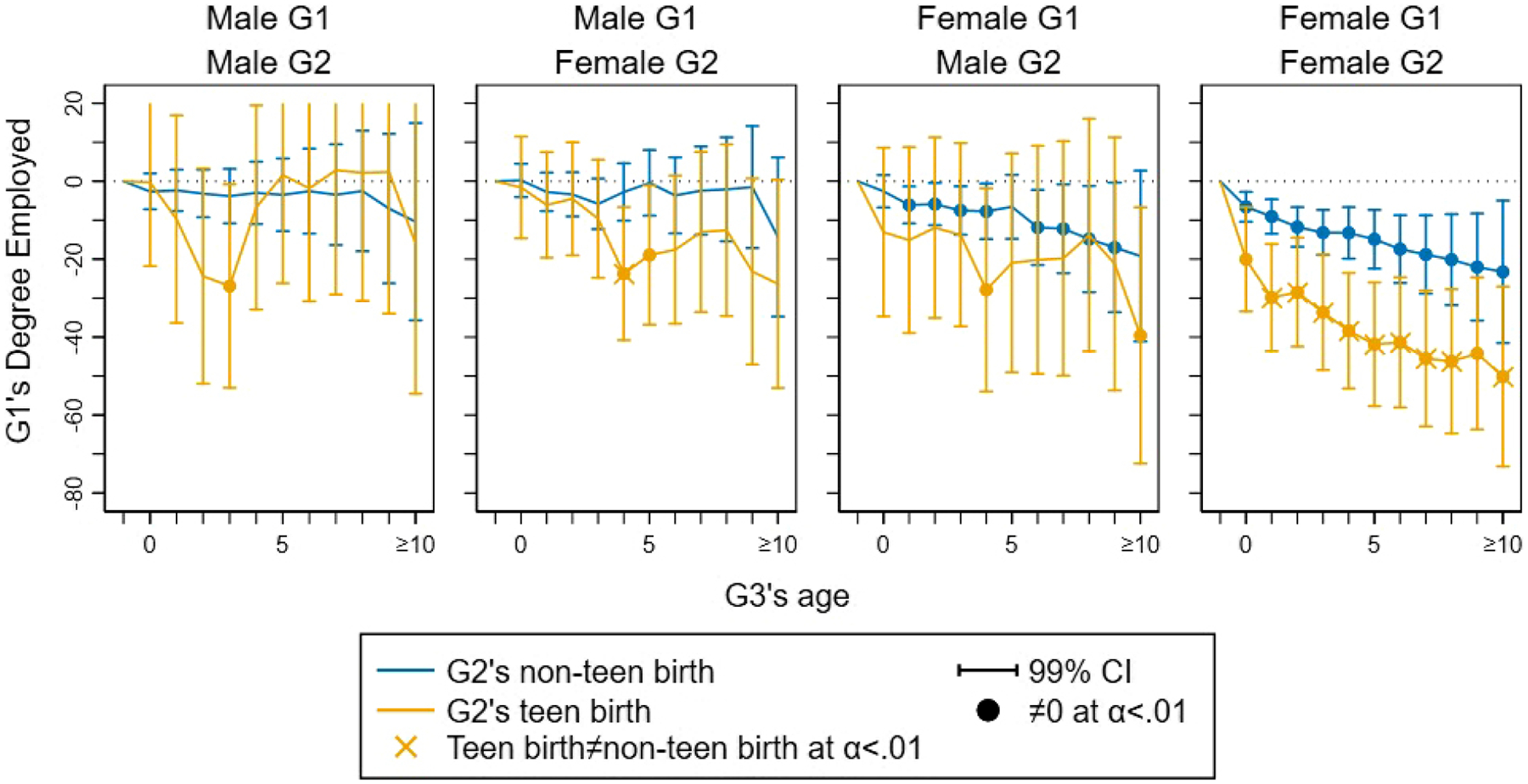
Trajectories of grandparenthood effects on G1’s degree of employment (1 to 1000) across G3’s first 10 years of life.

Figure 3 shows how the effect of grandparenthood on G1’s degree of employment evolves with the duration of grandparenthood. Because these models relax some constraints embedded in Equation (8), estimates are much less precise (larger confidence intervals). The left two panels for grandfathers show no dependable evidence for a sustained effect of grandparenthood on G1 grandfathers’ employment regardless of G2’s gender or teen birth. The right two panels for female G1 clearly show that the effect of grandparenthood on G1 grandmothers accumulates over time. This trend is evident also for G1 grandmothers of G2 sons, but is most precisely estimated (and hence consistently statistically significant) for G1 grandmothers of G2 daughters. The estimates of grandparenthood on G1 grandmothers’ employment are consistently stronger when the G2 daughter gives birth as a teenager rather than as an adult.

The same patterns hold for the evolution of grandparent effects on full-time employment and annual income (online supplement Figures B1 and B2). Negative grandparent effects accumulate over time and are strongest and most precisely estimated for G1 grandmothers of G2 daughters, especially when giving birth to G3 as teenagers. There is no evidence that grandparenthood effects abate over time.

### Grandparenthood Effects by G1’s Economic Resources

We assess effect modification in grandparenthood effects separately by tertiles of G1’s baseline individual labor income and baseline household income (when G2 was aged 13 to 15). Because of smaller samples, the estimates are often imprecise. Nevertheless, patterns are qualitatively similar across the three labor market outcomes, regardless of whether we stratify the analysis by G1’s labor income or household income.

**Figure 4:**
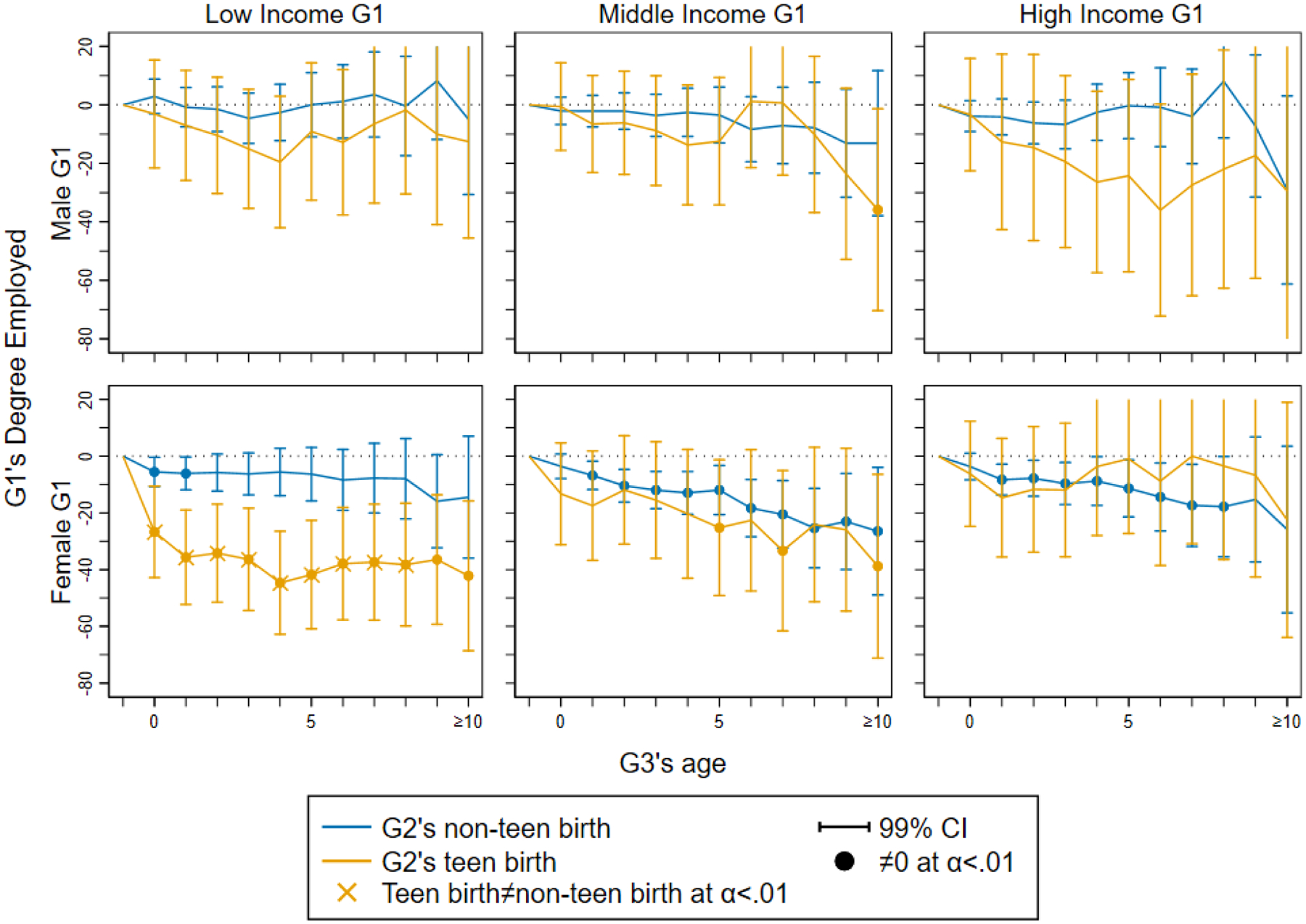
Trajectories of grandparenthood effects on G1’s degree of employment (1 to 1000) by G1’s baseline labor income at baseline (when G2 is aged 13 to 15) across G3’s first 10 years of life.

For illustration, Figure 4 shows how grandparenthood effects on G1’s degree employed vary with G1’s labor income at baseline (the remaining results are shown in online supplement Figures B3–B7). We find that G1 grandmothers with low baseline income experience a larger reduction in degree employed after G2’s teen birth than G1 grandmothers with middle or high income. By contrast, grandmotherhood effects from G2’s non-teen birth are of similar, modest size across all income groups (statistically significant for middle- and high-income grandmothers). Hence, hypothesis H3 about the modifying effect of grandparental economic resources on the grandparenthood effect is supported only for grandparenthood due to G2’s teen births but not for non-teen births. Grandparenthood effects on G1 grandfathers’ labor force outcomes by G1’s economic resources are generally small and negative, but too imprecisely estimated to allow interpretation.

## Discussion

Sociologists have long recognized the role of grandparents in the family support system. However, the consequences of grandparenthood for grandparent’s own labor market outcomes have received less attention. Using population-level multigenerational data in Denmark, this study shows that grandparenthood leads to a decrease in the employment and income of working-age grandparents, especially for G1 grandmothers of G2 daughters, and even more so when the G2 daughters give birth as teenagers. Additionally, we find that grandparenthood effects due to a daughter’s teen births appear to be larger for grandmothers with lower income.

Our results clarify the conditions under which parents’ caregiving obligations spill over to grandparents. First, we extend previous findings of heterogeneous grandparenthood effects by grandparents’ own gender (Backhaus and Barslund 2021; Frimmel et al. 2022; Kridahl 2017; Lumsdaine and Vermeer 2015; Rupert and Zanella 2018; Van Bavel and De Winter 2013) to show that the gender of the parent generation matters, too. Our findings—that the negative effects of grandparenthood on employment and income are especially pronounced when women become grandmothers due to their daughter’s birth—resonate with previous findings of active childcare support through maternal lines in Western societies (Di Gessa et al. 2016a; Geurts et al. 2015; Hank and Buber 2009; Thomese and Liefbroer 2013). Second, our findings imply that the effects of grandparenthood vary greatly by the caregiving needs of parents. The larger impact of grandmotherhood from a daughter’s teen birth may arise from limited parental resources of young mothers (Fergusson and Woodward 1999; Pogarsky et al. 2006) and their dependence on maternal kinship ties. Third, the larger impact of a daughter’s teen birth on grandmothers with lower income indicates the importance of grandparents’ capacity in managing the spillover of parenting.

That said, the magnitude of most grandparenthood effects on the labor market outcomes considered in this study is substantively small. Grandmotherhood effects amount to only a 1 percent to 2 percent decrease in employment and income from daughter’s non-teen birth. Not surprisingly, these grandmotherhood effects are smaller than the effect of motherhood in Denmark—a decrease of 7 percent in employment and 21 percent in income in the long run (Kleven et al. 2019). Furthermore, our estimates are also smaller than the 8 percent to 9 percent decrease in employment due to grandmotherhood in the United States, Austria, and Sweden (Frimmel et al. 2022; Kridahl 2017; Lumsdaine and Vermeer 2015). Our small estimates are consistent with previous findings that grandparents’ involvement in Denmark is limited because of universal public childcare and weak normative pressure for grandparenting (Albertini et al. 2007; Di Gessa et al. 2016a; Hank and Buber 2009). At the same time, however, a daughter’s teen birth has a substantively large impact on grandmothers’ labor force participation and annual income (−7 percent on both outcomes on average), even in Denmark.

The size of grandparenthood effects likely varies across institutional contexts and time. One might expect larger grandparenthood effects where public childcare support is more limited (e.g., the United States), gendered normative expectations for grandparenting are stronger (e.g., in East Asia), the importance of intergenerational support is heightened in the course of the second demographic transitions (e.g., due to smaller sibships, greater family instability, increased single parenthood) (Lesthaeghe 2010; Meyer 2014), and employer discrimination against older workers persists (van Dalen, Henkens, and Wang 2015; Roscigno et al. 2007).

Whether, and to what extent, the negative effects of grandmotherhood identified in this article should be labeled a “grandmotherhood penalty”—analogous to the “motherhood penalty” (Budig and England 2001; England et al. 2016; Gangl and Ziefle 2009)—is a question for further sociological research and normative debate. Presumably, some grandmothers reduce their labor force participation voluntarily—even eagerly—to spend more time with their daughters and grandchildren, with the expectation of emotional fulfillment from intergenerational bonding (Di Gessa, Glaser, and Tinker 2016b; Hughes et al. 2007). On the other hand, the effects of grandparenthood are gendered and unequally distributed. Considering that seemingly voluntary “opting-out” of mothers from professional careers often comes after painful negotiation in the family and workplace (Stone 2008), negative grandmotherhood effects may also reflect structural disadvantages of women in paid labor and housework. The present research establishes the size of these gendered causal grandparenthood effects but cannot disambiguate these competing interpretations.

We note several limitations. First, due to the nature of population register data, we cannot directly investigate the role of family norms, the value of intergenerational support, and the way grandmothers assess their changing roles after the initiation of grandmotherhood. These are promising topics for future research. Second, we cannot rule out that residual confounding biases our results, perhaps due to shared family norms that jointly favor G2 childbearing and G1’s gendered intergenerational support. That said, our models control for past labor force outcomes, which should proxy for the confounding effect of family norms and remediate the concern over bias. Hence, unless the extent of unobserved confounding differs markedly between grandmothers and grandfathers, and between teen mothers and non-teen mothers, our results indicate that the labor market consequences of grandparenthood are unequally distributed. In conclusion, this research indicates that, even in the most defamiliarized social policy regime, childbearing has multigenerational consequences for the labor force outcomes of grandparents, which are largely borne by women.

## Supplementary Material

supplement

## Figures and Tables

**Table 1:** Grandparenthood effects on G1’s labor market outcomes from R-MSMs.

	(1)	(2)	(3)	(4)	(5)	(6)
	Male G1	Male G1	Male G1	Female G1	Female G1	Female G1
Model and variable \ subsample		Male G2	Female G2		Male G2	Female G2
[a] Degree employed						
G2’s birth	−2.49 (1.43)	−3.05 (2.08)	−1.99 (1.98)	*−*8.89** (1.32)	−6.38* (1.93)	−10.92** (1.83)
G2’s birth × teen birth	−11.05 (4.68)	−6.03 (8.40)	−12.70 (5.62)	−25.97** (4.37)	−15.64 (8.02)	−27.33** (5.22)
Mean degree employed before G2’s birth	698.36	693.67	701.90	688.75	687.92	689.38
Mean degree employed before G2’s teen birth	647.21	668.44	640.14	556.26	580.87	548.30
[b] Full-time employment (%)						
G2’s birth	−0.28 (0.15)	−0.34 (0.22)	−0.24 (0.21)	−0.88** (0.14)	−0.68* (0.21)	−1.03** (0.20)
G2’s birth × teen birth	−1.09 (0.48)	−0.51 (0.87)	−1.29 (0.57)	−2.27** (0.45)	−1.64 (0.83)	−2.29** (0.54)
% full employment before G2’s birth	67.74	67.31	68.07	64.84	64.86	64.82
% full employment before G2’s teen birth	61.51	64.11	60.65	50.10	51.87	49.52
[c] Labor income (in DKK)						
G2’s birth	−1,060 (649)	−1,148 (950)	−1,011 (891)	−2,751** (483)	−1,898* (715)	−3,430** (658)
G2’s birth × teen birth	−2,814 (2,145)	−127 (3,576)	−3,608 (2,615)	−7,168** (1,446)	−3,885 (2,812)	−7,565** (1,704)
Mean labor income before G2’s birth	295,897	295,790	295,977	241,575	242,965	240,524
Mean labor income before G2’s teen birth	247,368	256,456	244,345	170,634	180,000	167,602
N(G1)	421,547	215,911	205,636	468,352	239,250	229,102
N(Gl-years)	5,646,121	2,888,532	2,757,589	6,534,538	3,334,053	3,200,485

*Note*: Cluster-robust standard errors at the individual level in parentheses. All models are weighted by stabilized inverse probability weights and include time-fixed covariates and time trends as described in the text (not shown).

* *p* < 0.01;

** *p* < 0.001.

## Data Availability

Because our data-use agreement prohibits direct sharing of our analytic data and original code, we share minimally redacted (removal of identifiers) analytic code and instructions to access our replication package at https://doi.org/10.7910/DVN/OV3IEK. Interested parties may apply to Statistics Denmark (https://www.dst.dk/en/TilSalg/Forskningsservice/Dataadgang/) for access to the replication package.

## Footnotes

1 Past research has used the gender of the firstborn child as an instrument for the timing of grandparenthood (Backhaus and Barslund 2021; Rupert and Zanella 2018) but did not investigate gender-specific labor market consequences of grandparenthood.

2 Although past research has estimated how grandparenthood effects on retirement vary with grandparents’ age (Kridahl 2017; Van Bavel and De Winter 2013), interactions with offspring’s fertility timing are unexplored.

3 Grandparenting by maternal grandmothers is not a historical or intercultural constant. For example, in many preindustrial and patrilineal societies, grandparenting fell to paternal grandmothers (Ko and Hank 2013; Zhang et al. 2019). This suggests that the observed trends of maternal grandmothering are rooted in Western family norms rather than evolutionary mechanisms as asserted by the grandmother hypothesis (Hawkes et al. 1998) or the paternity uncertainty hypothesis (Danielsbacka et al. 2011; Euler and Weitzel 1996).

4 All the statistics are retrieved from Statistics Denmark (https://www.statbank.dk/), accessed on May 22, 2024.

5 For example, if a G2 has a Danish G1 mother and a German G1 father, then the G1 mother is included as a subject in the analysis, but the G1 father is not.

6 Most excluded G1s were self-employed; negative income reflects business losses.

7 Although Danish families are small and siblings are tightly spaced, we therefore miss some younger and older siblings of the G2s in our analysis.

8 Alternatively, we could have defined grandparenthood as the birth of G1’s first G3 from any G2. This, however, would greatly have complicated prospectively controlling for the all G2s’ time-varying characteristics. The difference between these two definitions of grandparenthood is small. Our definition of grandparenthood coincides with the birth of 84.7 percent of G1’s first G3 from any G2 (i.e., 320,086 out of 377,917 first G3 births in G1’s lineage).

9 The income threshold roughly equals the average annual income in 2012 (=288,684 DKK).

10 Identification in R-MSM hinges on the correct specification of both the outcomes model and the model for the weights. We have extensively explored alternative specifications for the weights and found that the estimated treatment effects were stable across weights models but decreased when allowing predictors to interact with G2’s age. In order to impose the fewest parametric assumptions in the estimation of the weights, we report this most flexibly interacted model.

11 The numerator “stabilizes” the inverse probability weights in the sense of reducing the variance of the outcomes model (Hernán et al. 2002). Estimators are consistent even if the numerator is misspecified as long as the denominator is correctly specified (Robins 1999).

12 We impute less than 0.1 percent of G1’s and 1 percent of G2’s labor market histories when only one or two consecutive missing values exist in a panel, based on the assumption of linear growth. We also impute less than 0.1 percent of G1’s and 9 percent of G2’s educational attainment from valid past and future observations. We have more missing values for G2’s education because years of education are only available from age 15. In this case, we assume that every G2 starts education at age six. The remaining missing values in labor market histories and educational attainment are adjusted using the attrition weights.

13 We model attrition as a function of twice-lagged information (t-2) because missingness in covariates (t-1) leads to attrition in the subsequent period (t).
