# Supplementary material for: Labor Market Consequences of Grandparenthood
Source: Sociol Sci. Author manuscript; Available in PMC 2025 May 22. (PMC12097765; doi:10.15195/v11.a22)
Supplement: supplement [file NIHMS2078355-supplement-supplement.pdf]

sociological science

Supplement to:

Joo, Won-tak, Felix Elwert, and Martin D. Munk. 2024. "Labor Market Consequences of Grandparenthood" Sociological Science 11: 600-625.

A. Appendix Tables

Table A1. List of variables

Category		Variable
Y: G1's labor market outcomes		Degree employed, full-time employment, labor income
D: G1's grandparenthood		First G2 has had a child, years since first G2 has had a child (= G3's age)
F: Time-fixed covariates		G1's gender, first G2's gender, G1's any teen birth
S: Time trends		First G2' age (t=1 when G2 is 13), G1's age, calendar year
V: Time-varying covariates for	G1	Work experience, years of education, current educational enrollment, comorbidity index, days in hospital, any mental health contact, # total births, partnered, partner full-time employed, partner with labor income ≥300k, partner with any chronic disease, partner hospitalized ≥5 days, partner with any mental health contact
	First G2	Degree employed, labor income, work experience, partnered, years of education, current educational enrollment, comorbidity index, days in hospital, any mental health contact
	All G2*	# G2, # G3, # female G2, # full-time employed G2, # G2 with labor income ≥300k, # partnered G2, # G2 with ≥16 years of education, # G2 currently enrolled in school, # G2 with any chronic disease, # G2 hospitalized ≥5 days, # G2 with any mental health contact
	Household	# Household members, # G2 in household, # household members aged <18, household gross income, any public assistance

* The covariates for "all G2" refer to measures about G1's first four G2, i.e. all G2 included in the data. # means "number of."

Table A2. Descriptive statistics

Variable	Male G1		Male G1 Male G2		Male G1 Female G2		Female G1		Female G1 Male G2		Female G1 Female G2	
First G2's age	13	25	13	25	13	25	13	25	13	25	13	25
G1's labor market outcomes												
Degree employed	755.25	716.17	754.67	714.95	755.86	717.44	686.72	712.65	685.74	712.33	687.74	712.99
Full-time employment	0.73	0.70	0.73	0.69	0.73	0.70	0.60	0.67	0.60	0.67	0.61	0.67
Labor income (thousand 2012 DKK)	308.70	308.40	308.39	307.73	309.02	309.10	219.83	252.48	219.57	252.49	220.10	252.48
G1's grandparenthood												
First G2 has had a child	0.00	0.16	0.00	0.11	0.00	0.21	0.00	0.16	0.00	0.11	0.00	0.21
First G2's teen birth	0.00	0.02	0.00	0.01	0.00	0.03	0.00	0.02	0.00	0.01	0.00	0.04
Time-fixed covariates												
G1's any teen birth	0.03	0.04	0.03	0.04	0.03	0.04	0.12	0.14	0.12	0.14	0.12	0.14
Time trends												
G1's age	41.78	53.38	41.77	53.37	41.79	53.39	39.44	51.27	39.43	51.27	39.45	51.27
Calendar year	1996.41	2005.61	1996.43	2005.61	1996.40	2005.61	1996.28	2005.59	1996.29	2005.59	1996.26	2005.60
Time-varying covariates: G1												
Work experience (years)	12.77	20.07	12.77	20.05	12.76	20.10	10.09	17.26	10.10	17.24	10.08	17.27
Years of education	12.33	12.77	12.34	12.78	12.33	12.76	11.99	12.48	11.99	12.48	11.98	12.48
Current educational enrollment	0.01	0.00	0.01	0.00	0.01	0.00	0.03	0.02	0.03	0.02	0.03	0.02
Comorbidity index	0.01	0.08	0.01	0.08	0.01	0.08	0.01	0.07	0.01	0.08	0.01	0.07
Days in hospital	0.42	0.62	0.41	0.62	0.43	0.62	0.58	0.56	0.57	0.55	0.58	0.56
Any mental health contact	0.01	0.01	0.01	0.01	0.01	0.01	0.01	0.01	0.01	0.01	0.01	0.01
# Total births	2.24	2.36	2.25	2.36	2.24	2.35	2.26	2.34	2.27	2.35	2.25	2.33
Marital status: partnered	0.74	0.76	0.74	0.76	0.74	0.76	0.73	0.72	0.73	0.72	0.73	0.72
Marital status: divorced	0.12	0.15	0.11	0.15	0.12	0.15	0.12	0.17	0.12	0.17	0.12	0.17
Marital status: widowed	0.00	0.01	0.00	0.01	0.00	0.01	0.01	0.03	0.01	0.03	0.01	0.03
Marital status: never married	0.14	0.08	0.14	0.08	0.14	0.07	0.14	0.08	0.14	0.08	0.14	0.08
Partner full-time employed: yes	0.46	0.41	0.46	0.41	0.46	0.42	0.37	0.44	0.37	0.44	0.37	0.44
Partner full-time employed: no	0.52	0.58	0.52	0.58	0.52	0.58	0.62	0.55	0.62	0.55	0.62	0.55
Partner full-time employed: missing	0.01	0.01	0.01	0.01	0.01	0.01	0.02	0.01	0.02	0.01	0.02	0.01
Partner with labor income ≥300k	0.24	0.36	0.24	0.36	0.24	0.36	0.53	0.50	0.53	0.50	0.53	0.50
Partner with any chronic disease	0.01	0.04	0.01	0.04	0.01	0.03	0.01	0.04	0.01	0.04	0.01	0.04
Partner hospitalized ≥5 days	0.03	0.02	0.03	0.02	0.03	0.02	0.02	0.02	0.02	0.02	0.02	0.02
Partner with any mental health contact	0.00	0.01	0.00	0.01	0.00	0.01	0.00	0.00	0.00	0.00	0.00	0.00
Time-varying covariates: First G2												
Degree employed	0.00	543.33	0.00	594.58	0.00	489.87	0.00	536.25	0.00	585.56	0.00	485.01

Labor income (thousand 2012 DKK)	2.59	175.66	3.07	204.39	2.09	145.69	2.58	173.60	3.05	201.49	2.09	144.61
Work experience (years)	0.00	3.79	0.00	4.35	0.00	3.21	0.00	3.74	0.00	4.28	0.00	3.18
Partnered	0.00	0.05	0.00	0.03	0.00	0.08	0.00	0.05	0.00	0.03	0.00	0.08
Years of education	6.00	12.64	6.00	12.63	6.00	12.65	6.00	12.62	6.00	12.61	6.00	12.64
Current educational enrollment	1.00	0.40	1.00	0.33	1.00	0.47	1.00	0.40	1.00	0.34	1.00	0.47
Comorbidity index	0.01	0.02	0.01	0.01	0.00	0.02	0.01	0.02	0.01	0.01	0.00	0.02
Days in hospital	0.19	0.43	0.19	0.24	0.20	0.63	0.19	0.43	0.19	0.24	0.20	0.63
Any mental health contact	0.00	0.02	0.00	0.01	0.00	0.02	0.00	0.02	0.00	0.01	0.00	0.02
Time-varying covariates: Up-to-four G2												
# G2	1.61	1.75	1.61	1.76	1.60	1.74	1.60	1.72	1.61	1.73	1.59	1.71
# G3	0.00	0.23	0.00	0.16	0.00	0.31	0.00	0.23	0.00	0.16	0.00	0.31
# Female G2	0.78	0.85	0.30	0.37	1.29	1.36	0.78	0.84	0.29	0.35	1.29	1.35
# G2 full-time employed	0.00	0.65	0.00	0.72	0.00	0.58	0.00	0.63	0.00	0.70	0.00	0.56
# G2 with labor income ≥300k	0.00	0.26	0.00	0.36	0.00	0.15	0.00	0.25	0.00	0.35	0.00	0.15
# G2 partnered	0.00	0.06	0.00	0.04	0.00	0.09	0.00	0.06	0.00	0.04	0.00	0.08
# G2 with ≥16 years of education	0.00	0.06	0.00	0.04	0.00	0.07	0.00	0.06	0.00	0.04	0.00	0.07
# G2 currently enrolled in school	1.55	0.84	1.55	0.78	1.54	0.91	1.55	0.83	1.55	0.77	1.54	0.89
# G2 with any chronic disease	0.01	0.02	0.01	0.02	0.01	0.02	0.01	0.02	0.01	0.02	0.01	0.02
# G2 hospitalized ≥5 days	0.02	0.03	0.02	0.02	0.02	0.05	0.02	0.03	0.02	0.02	0.02	0.05
# G2 with any mental health contact	0.00	0.03	0.00	0.03	0.00	0.03	0.00	0.03	0.01	0.03	0.00	0.03
Time-varying covariates: Household												
# Household members	3.60	2.43	3.61	2.48	3.58	2.38	3.86	2.41	3.87	2.45	3.86	2.35
# G2 in household	1.25	0.43	1.26	0.48	1.24	0.38	1.53	0.48	1.53	0.53	1.53	0.43
# Household members aged <18	1.60	0.32	1.61	0.33	1.59	0.32	1.87	0.32	1.87	0.33	1.87	0.32
Household income (thousand 2012 DKK)	683.87	792.49	683.92	804.17	683.82	780.31	696.95	795.89	696.01	808.72	697.92	782.56
Any public assistance	0.43	0.36	0.43	0.37	0.43	0.35	0.44	0.36	0.44	0.37	0.44	0.35
N	421547	262838	215911	134196	205636	128642	468352	308024	239250	156968	229102	151056

Note: Author's calculation, data assembled from Danish Population Registries, as described in the body of the text.

Table A3. Weights for treatment and attrition

Subsample and weight	Mean	SD	Percentiles				
			1st	25th	50th	75th	99th
Male G1 (N=5,646,121)							
Weights for treatment (WT)	1.001	0.289	0.389	0.981	1.000	1.001	1.858
Weights for attrition (WA)	1.007	7.773	0.923	0.990	0.998	1.002	1.118
Final weights (=WT × WA)	1.009	8.926	0.389	0.972	0.998	1.003	1.892
Male G1, Male G2 (N =2,888,532)							
Weights for treatment (WT)	1.000	0.215	0.471	0.988	1.000	1.001	1.721
Weights for attrition (WA)	1.009	9.193	0.923	0.990	0.998	1.002	1.119
Final weights (=WT × WA)	1.011	10.607	0.470	0.979	0.998	1.003	1.753
Male G1, Female G2 (N=2,757,589)							
Weights for treatment (WT)	1.002	0.351	0.347	0.970	0.999	1.001	1.975
Weights for attrition (WA)	1.004	5.932	0.923	0.990	0.998	1.002	1.116
Final weights (=WT × WA)	1.007	6.730	0.346	0.962	0.997	1.004	2.003
Female G1 (N=6,534,538)							
Weights for treatment (WT)	1.001	0.296	0.391	0.980	1.000	1.001	1.878
Weights for attrition (WA)	1.015	20.984	0.950	0.994	0.999	1.001	1.076
Final weights (=WT × WA)	1.045	63.678	0.391	0.975	0.999	1.002	1.902
Female G1, Male G2 (N=3,334,053)							
Weights for treatment (WT)	1.000	0.217	0.468	0.987	1.000	1.001	1.747
Weights for attrition (WA)	1.002	1.081	0.950	0.994	0.999	1.001	1.077
Final weights (=WT × WA)	1.002	1.247	0.467	0.982	0.999	1.002	1.773
Female G1, Female G2 (N=3,200,485)							
Weights for treatment (WT)	1.003	0.361	0.350	0.969	1.000	1.002	2.005
Weights for attrition (WA)	1.029	29.964	0.950	0.994	0.999	1.001	1.075
Final weights (=WT × WA)	1.089	90.980	0.350	0.964	0.998	1.003	2.030

Note: Table shows descriptive statistics for untrimmed weights. For the analysis, we trimmed 658 weights ≥14 to 14.0 for the R-MSM outcomes models.

Table A4. Grandparenthood effects on G1's degree employed

Model and variable \ subsample	(1) Male G1	(2) Male G1 Male G2	(3) Male G1 Female G2	(4) Female G1	(5) Female G1 Male G2	(6) Female G1 Female G2
Regression models						
[a] Unadjusted beyond time trends						
G2's birth	-26.06**	-20.51**	-33.68**	-37.13**	-25.67**	-50.34**
	(1.48)	(2.19)	(2.02)	(1.36)	(2.02)	(1.85)
G2's birth × teen birth	-101.67**	-80.84**	-106.77**	-152.30**	-130.62**	-155.67**
	(4.67)	(9.14)	(5.47)	(4.44)	(8.78)	(5.19)
[b] (model a) + time-fixed covariates						
G2's birth	-5.97**	-4.59	-7.22**	-11.05**	-7.09**	-14.63**
	(1.26)	(1.86)	(1.70)	(1.16)	(1.73)	(1.58)
G2's birth × teen birth	-23.63**	-10.92	-27.12**	-45.62**	-32.38**	-48.45**
	(3.66)	(6.91)	(4.34)	(3.59)	(7.01)	(4.23)
[c] (model b) + time-varying covariates						
G2's birth	-0.13	0.07	-0.30	-1.36*	-0.23	-2.27**
	(0.46)	(0.68)	(0.62)	(0.39)	(0.58)	(0.54)
G2's birth × teen birth	-1.82	-1.43	-2.13	-0.07	2.15	-0.78
	(0.93)	(1.70)	(1.12)	(0.82)	(1.53)	(0.98)
[d] Individual fixed effects + time-varying covariates						
G2's birth	0.18	0.67	-0.47	-1.57	-0.88	-2.25
	(0.75)	(1.14)	(1.00)	(0.65)	(0.97)	(0.87)
G2's birth × teen birth	-9.80**	-8.83	-9.58*	-8.04**	-3.94	-9.24**
	(2.36)	(4.39)	(2.82)	(2.20)	(4.27)	(2.59)
Marginal structural models						
[e] (model b) + weights						
G2's birth	-2.49	-3.05	-1.99	-8.89**	-6.38*	-10.92**
	(1.43)	(2.08)	(1.98)	(1.32)	(1.93)	(1.83)
G2's birth × teen birth	-11.05	-6.03	-12.70	-25.97**	-15.64	-27.33**
	(4.68)	(8.40)	(5.62)	(4.37)	(8.02)	(5.22)
Mean(degree employed) before G2's birth	698.36	693.67	701.90	688.75	687.92	689.38
Mean(degree employed) before G2's teen-birth	647.21	668.44	640.14	556.26	580.87	548.30
N(G1)	421547	215911	205636	468352	239250	229102
N(G1-years)	5646121	2888532	2757589	6534538	3334053	3200485

Note: Cluster-robust standard errors at the individual level in parentheses. Coefficients and standard errors for time-fixed covariates and time trends are not shown. All models are weighted by stabilized inverse probability weights. * p<0.01; ** p<0.001.

Table A5. Grandparenthood effects on G1's full-time employment

Model and variable \ subsample	(1) Male G1	(2) Male G1 Male G2	(3) Male G1 Female G2	(4) Female G1	(5) Female G1 Male G2	(6) Female G1 Female G2
Regression models						
[a] Unadjusted beyond time trends						
G2's birth	-2.82**	-2.19**	-3.66**	-3.84**	-2.72**	-5.13**
	(0.15)	(0.23)	(0.21)	(0.15)	(0.22)	(0.20)
G2's birth × teen birth	-10.84**	-8.64**	-11.37**	-15.23**	-13.26**	-15.51**
	(0.48)	(0.95)	(0.57)	(0.46)	(0.91)	(0.54)
[b] (model a) + time-fixed covariates						
G2's birth	-0.67**	-0.50	-0.83**	-1.12**	-0.79**	-1.41**
	(0.13)	(0.19)	(0.18)	(0.13)	(0.19)	(0.17)
G2's birth × teen birth	-2.52**	-1.19	-2.88**	-4.24**	-3.19**	-4.46**
	(0.38)	(0.72)	(0.45)	(0.37)	(0.73)	(0.44)
[c] (model b) + time-varying covariates						
G2's birth	-0.02	0.06	-0.07	-0.13*	-0.04	-0.20*
	(0.06)	(0.08)	(0.08)	(0.05)	(0.07)	(0.07)
G2's birth × teen birth	-0.08	-0.03	-0.14	0.14	0.30	0.08
	(0.12)	(0.22)	(0.14)	(0.11)	(0.20)	(0.13)
[d] Individual fixed effects + time-varying covariates						
G2's birth	-0.02	0.03	-0.08	-0.15	-0.14	-0.16
	(0.08)	(0.13)	(0.11)	(0.08)	(0.12)	(0.11)
G2's birth × teen birth	-0.82*	-0.67	-0.81	-1.22**	-0.54	-1.43**
	(0.27)	(0.51)	(0.32)	(0.27)	(0.52)	(0.32)
Marginal structural models						
[e] (model b) + weights						
G2's birth	-0.28	-0.34	-0.24	-0.88**	-0.68*	-1.03**
	(0.15)	(0.22)	(0.21)	(0.14)	(0.21)	(0.20)
G2's birth × teen birth	-1.09	-0.51	-1.29	-2.27**	-1.64	-2.29**
	(0.48)	(0.87)	(0.57)	(0.45)	(0.83)	(0.54)
%(full employment) before G2's birth	67.74	67.31	68.07	64.84	64.86	64.82
%(full employment) before G2's teen-birth	61.51	64.11	60.65	50.10	51.87	49.52
N(G1)	421547	215911	205636	468352	239250	229102
N(G1-years)	5646121	2888532	2757589	6534538	3334053	3200485

Note: Cluster-robust standard errors at the individual level in parentheses. Coefficients and standard errors for time-fixed covariates and time trends are not shown. All models are weighted by stabilized inverse probability weights. The outcome is multiplied by 100. * p<0.01; ** p<0.001.

Table A6. Grandparenthood effects on G1's labor income

Model and variable \ subsample	(1) Male G1	(2) Male G1 Male G2	(3) Male G1 Female G2	(4) Female G1	(5) Female G1 Male G2	(6) Female G1 Female G2
Regression models						
[a] Unadjusted beyond time trends						
G2's birth	-22026**	-17288**	-27964**	-19849**	-14585**	-25886**
	(703)	(1042)	(963)	(529)	(786)	(720)
G2's birth × teen birth	-57819**	-48217**	-59441**	-58737**	-51942**	-59241**
	(2036)	(4090)	(2369)	(1566)	(3123)	(1827)
[b] (model a) + time-fixed covariates						
G2's birth	-5216**	-3682**	-6593**	-5272**	-3737**	-6634**
	(569)	(845)	(772)	(425)	(632)	(574)
G2's birth × teen birth	-10156**	-4269	-11494**	-13254**	-9727**	-13854**
	(1554)	(2978)	(1837)	(1217)	(2426)	(1425)
[c] (model b) + time-varying covariates						
G2's birth	-18	270	-270	-537**	-41	-873**
	(166)	(249)	(225)	(118)	(175)	(161)
G2's birth × teen birth	-315	58	-300	120	532	87
	(316)	(615)	(375)	(226)	(433)	(272)
[d] Individual fixed effects + time-varying covariates						
G2's birth	-378	-154	-580	-1273**	-809*	-1549**
	(277)	(417)	(373)	(190)	(286)	(257)
G2's birth × teen birth	-3285**	-2965	-3334*	-2302**	-632	-2600**
	(826)	(1605)	(975)	(594)	(1196)	(696)
Marginal structural models						
[e] (model b) + weights						
G2's birth	-1060	-1148	-1011	-2751**	-1898*	-3430**
	(649)	(950)	(891)	(483)	(715)	(658)
G2's birth × teen birth	-2814	-127	-3608	-7168**	-3885	-7565**
	(2145)	(3576)	(2615)	(1446)	(2812)	(1704)
Mean(labor income) before G2's birth	295897	295790	295977	241575	242965	240524
Mean(labor income) before G2's teen-birth	247368	256456	244345	170634	180000	167602
N(G1)	421547	215911	205636	468352	239250	229102
N(G1-years)	5646121	2888532	2757589	6534538	3334053	3200485

Note: Cluster-robust standard errors at the individual level in parentheses. Coefficients and standard errors for time-fixed covariates and time trends are not shown. All models are weighted by stabilized inverse probability weights. * p<0.01; ** p<0.001.

B. Appendix Figures

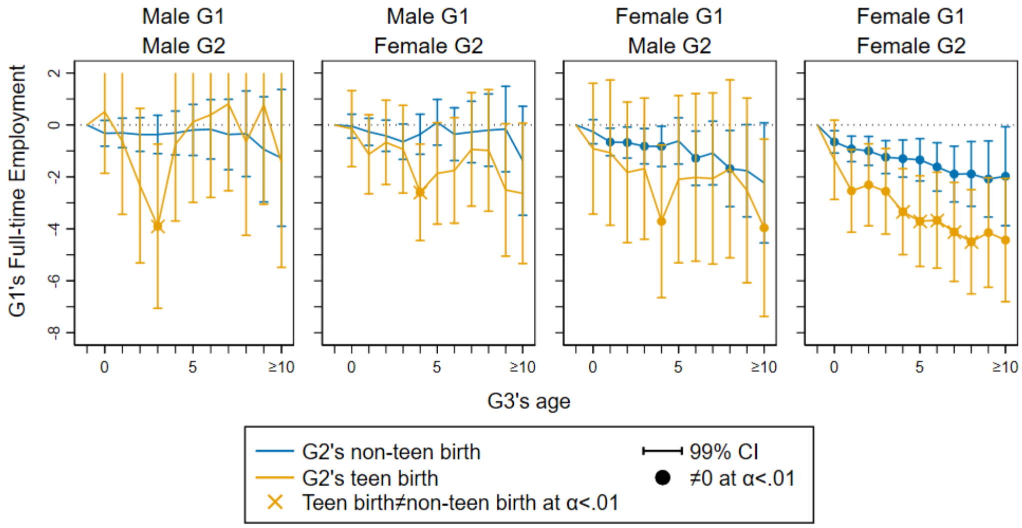

Figure B1. Trajectories of grandparenthood effects on G1's full-time employment across G3’s first 10 years of life.

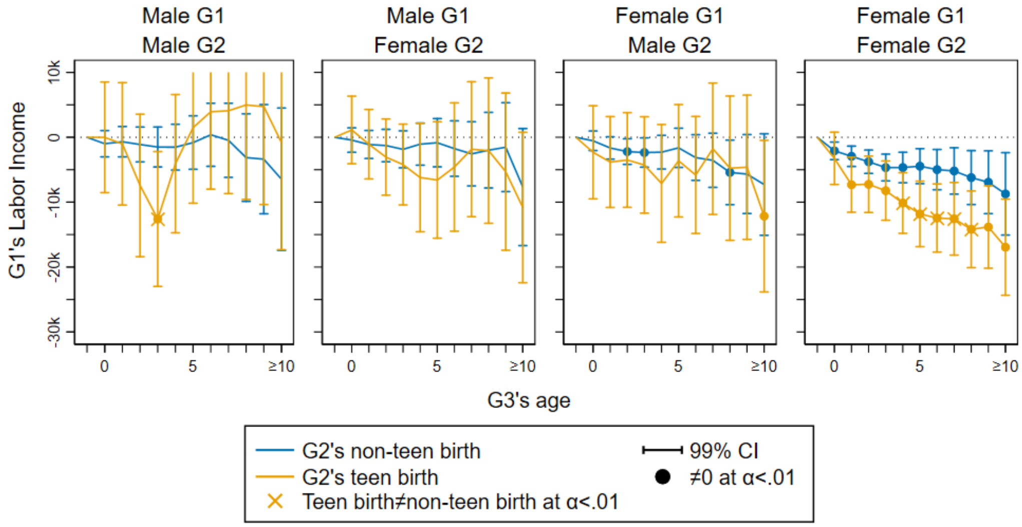

Figure B2. Trajectories of grandparenthood effects on G1's labor income across G3's first 10 years of life.

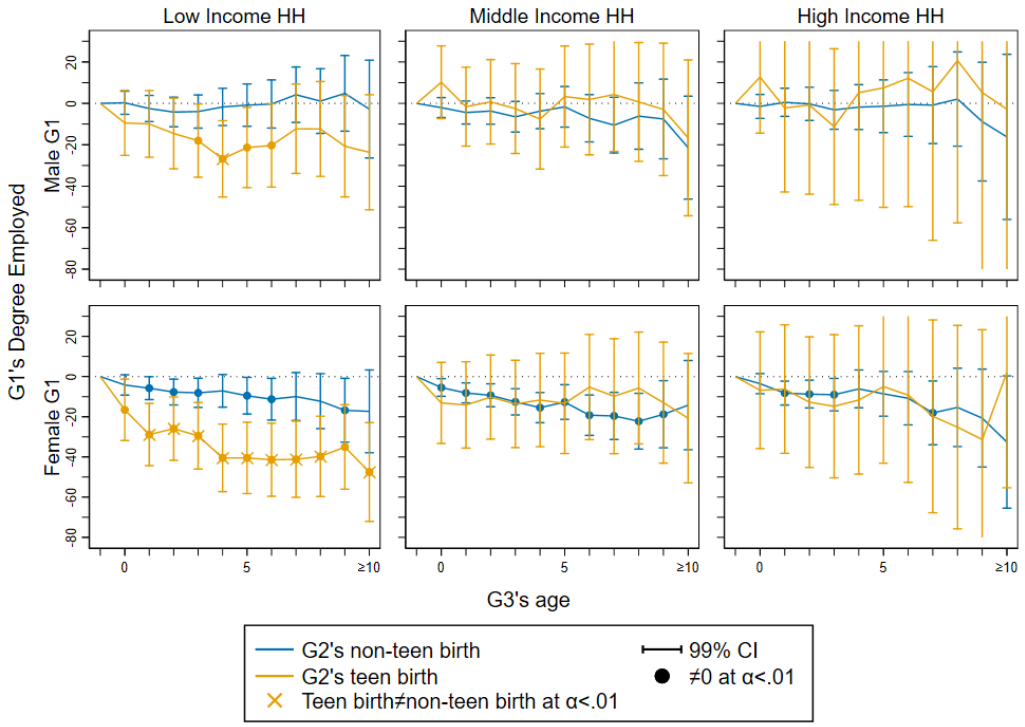

Figure B3. Trajectories of grandparenthood effects on G1's degree of employment (1-1000) by G1's household income at baseline (when G2 is aged 13-15) across G3's first 10 years of life.

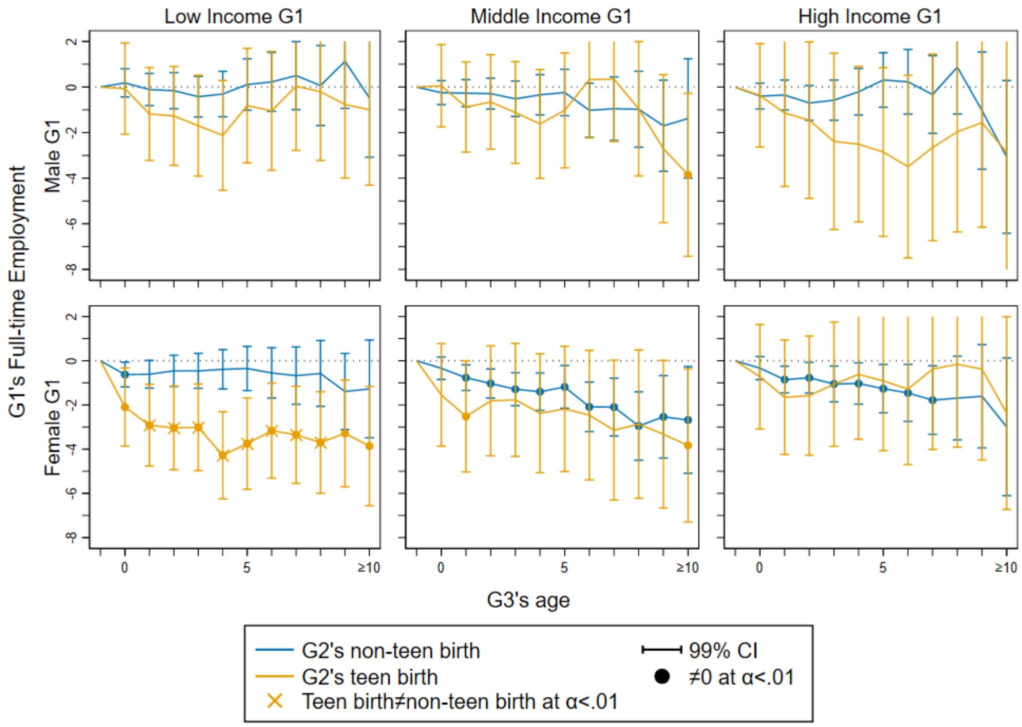

Figure B4. Trajectories of grandparenthood effects on G1's full-time employment by G1's labor income at baseline (when G2 is aged 13-15) across G3's first 10 years of life.

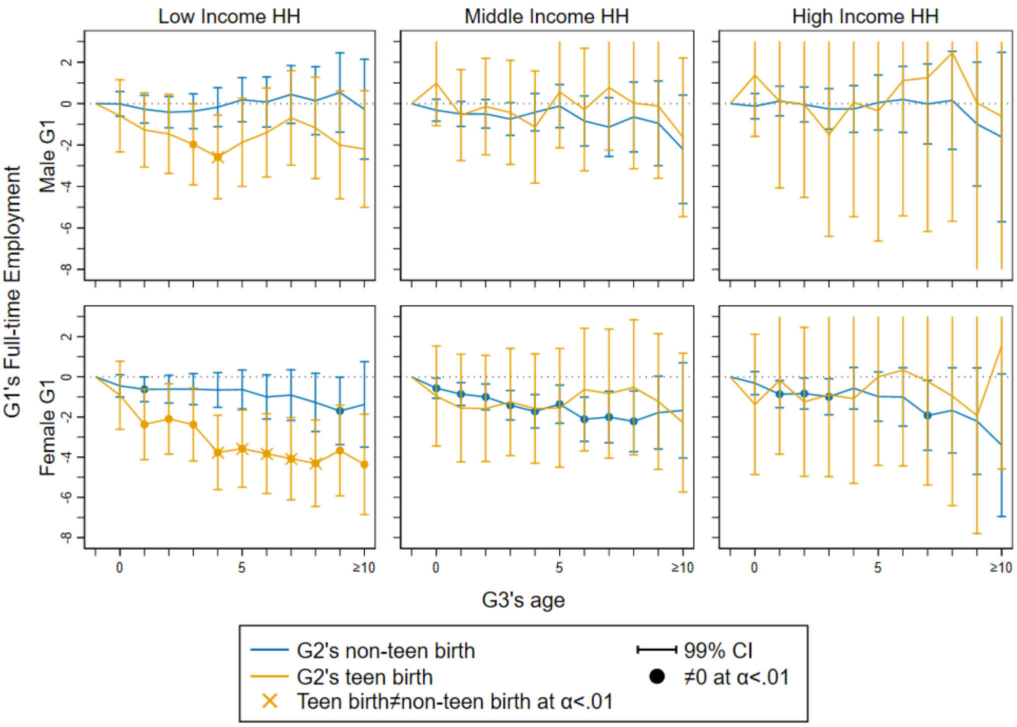

Figure B5. Trajectories of grandparenthood effects on G1's full-time employment by G1's household income at baseline (when G2 is aged 13-15) across G3's first 10 years of life.

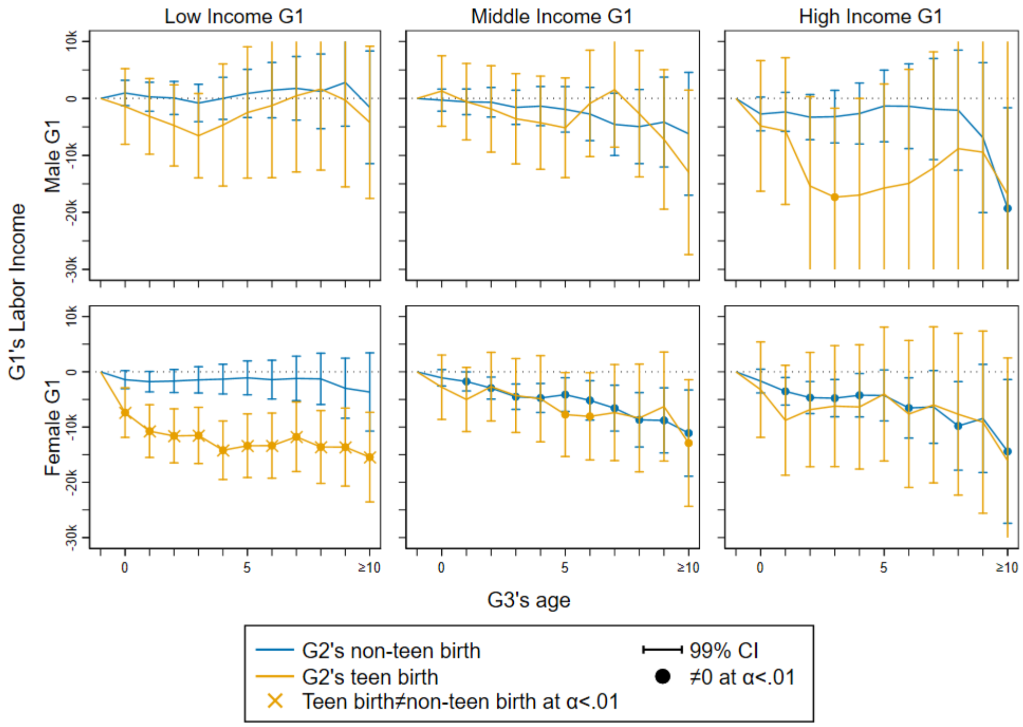

Figure B6. Trajectories of grandparenthood effects on G1's labor income by G1's labor income at baseline (when G2 is aged 13-15) across G3's first 10 years of life.

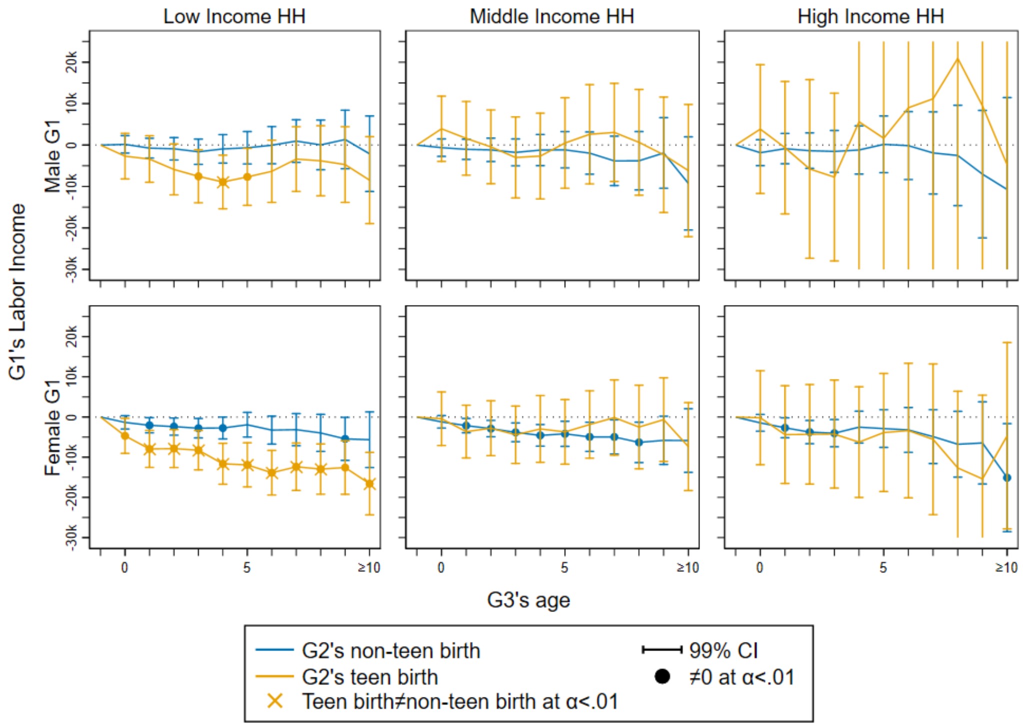

Figure B7. Trajectories of grandparenthood effects on G1's labor income by G1's household income at baseline (when G2 is aged 13-15) across G3's first 10 years of life.
